# 1752. Seasonal Human Coronavirus in Pediatric Patients enrolled by the New Vaccine Surveillance Network, 2016-2020

**DOI:** 10.1093/ofid/ofad500.1583

**Published:** 2023-11-27

**Authors:** Samar Musa, Xueyan Zhang, G K Balasubramani, Natasha B Halasa, Laura S Stewart, Jennifer E Schuster, Rangaraj Selvarangan, Geoffrey A Weinberg, Peter G Szilagyi, Eileen J Klein, Janet A Englund, Vasanthi Avadhanula, Pedro A Piedra, Christina M Quigley, Mary A Staat, Ariana Perez, Heidi L Moline, John V Williams, Marian G Michaels

**Affiliations:** University of Pittsburgh, Pittsburgh, Pennsylvania; University of Pittsburgh, Pittsburgh, Pennsylvania; University of Pittsburgh, Pittsburgh, Pennsylvania; Vanderbilt University Medical Center, Nashville, Tennessee; Vanderbilt University Medical Center, Nashville, Tennessee; Children’s Mercy Kansas City, Kansas City, Missouri; Children’s Mercy Kansas City, Kansas City, Missouri; University of Rochester School of Medicine & Dentistry, Rochester, NY; UCLA School of Medicine, Agoura Hills, California; University of Washington School of Medicine, Seattle, Washington; Seattle Children’s Hospital, Seattle, Washington; Baylor College of Medicine, Houston, Texas; Baylor College of Medicine, Houston, Texas; Cincinnati Children's Hospital Medical Center, Cincinnati, Ohio; Cincinnati Children’s Hospital Medical Center, Cincinnati, Ohio; CDC, Avondale Estates, Georgia; Centers for Disease Control and Prevention, Atlanta, Georgia; University of Pittsburgh, Pittsburgh, Pennsylvania; UPMC Children's Hospital of Pittsburgh, Pittsburgh, Pennsylvania

## Abstract

**Background:**

The COVID-19 pandemic highlighted the potential pathogenicity of betacoronaviruses. This elucidates the need to further investigate human coronaviruses (HCoV), which includes both alpha and betacoronaviruses, to better understand their seasonality and severity among children.

**Methods:**

The New Vaccine Surveillance Network (NVSN) enrolled children at 7 pediatric medical centers in the United States seen at the emergency department (ED) or hospitalized with acute respiratory infection (ARI), during 12/1/2016–9/28/2020. Respiratory specimens were collected and tested using real-time reverse transcription polymerase-chain reaction assays. The frequency of seasonal HCoV (HCoV-229E, HCoV-HKU1, HCoV-NL63, and HCoV-OC43) detections was measured and symptoms were reported by parent interview. Descriptive statistics were performed to compare HCoV-positive inpatients to those seen in the ED. SARS-CoV-2 infections were excluded.

**Results:**

Of 34,455 children enrolled, 925 (2.7%) were positive for HCoV, of which 398 (43%) were inpatients and (57%) were ED patients (Table). 346 detections (37.4%) were HCoV-OC43, 298 (32.3%) HCoV-NL63, 186 (20.2%) HCoV-HKU1, and 107 (11.6%) HCoV-229E. More than one coronavirus was detected in 12 children (1.3%). HCoV were primarily detected between October and April of each season; 1 to 2 types dominated each year but not consecutively (Figure). Among HCoV-positive children, 527 (57.0%) received their highest level of care in the ED while 398 (43.0%) were hospitalized. HCoV-229E was the most common HCoV detected among inpatients (57.0%), followed by HCoV-HKU1-(45.2%); HCoV-OC43- (40.8%); and HCoV-NL63 (38.9%). Among those positive for a HCoV age, race, and insurance status were significantly associated with hospitalization.

**Figure:**
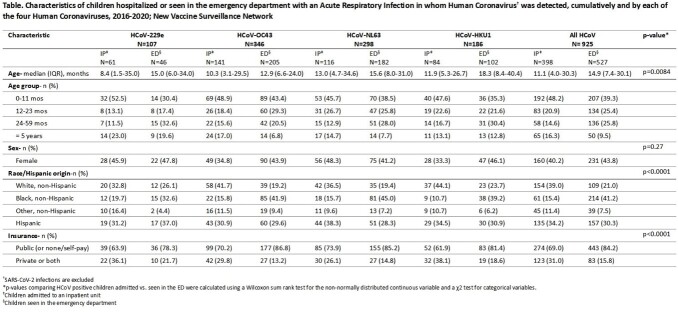

**Figure:**
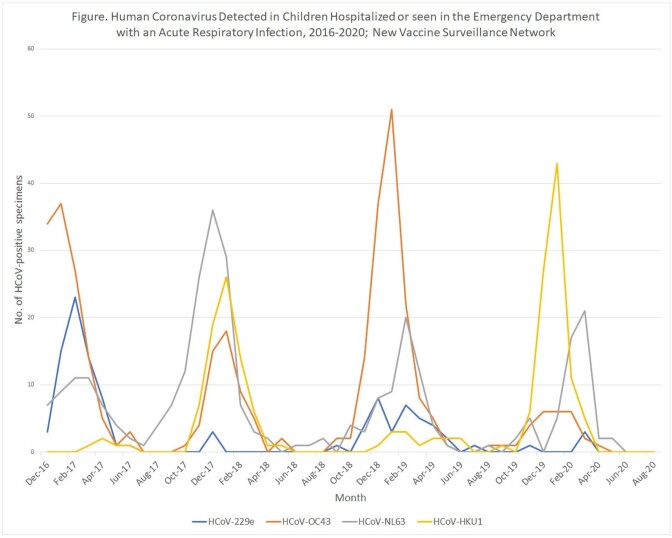

**Conclusion:**

HCoV followed a winter-season circulation pattern, but with variation in the predominant type across years. HCoV-OC43 accounted for the largest number of HCoV infections whereas HCoV-229E had the highest proportion hospitalized. Further surveillance is required to evaluate the impact of the emergent circulation of SARS-CoV-2 on seasonal HCoVs.

**Disclosures:**

**Natasha B. Halasa, MD, MPH**, Merck: Grant/Research Support|Quidell: Grant/Research Support|Quidell: donation of kits|Sanofi: Grant/Research Support|Sanofi: vaccine support **Rangaraj Selvarangan, BVSc, PhD, D(ABMM), FIDSA, FAAM**, Abbott: Honoraria|Altona Diagnostics: Grant/Research Support|Baebies Inc: Advisor/Consultant|BioMerieux: Advisor/Consultant|BioMerieux: Grant/Research Support|Bio-Rad: Grant/Research Support|Cepheid: Grant/Research Support|GSK: Advisor/Consultant|Hologic: Grant/Research Support|Lab Simply: Advisor/Consultant|Luminex: Grant/Research Support **Geoffrey A. Weinberg, MD**, Merck & Co: Honoraria **Janet A. Englund, MD**, Ark Biopharma: Advisor/Consultant|AstraZeneca: Advisor/Consultant|AstraZeneca: Grant/Research Support|GlaxoSmithKline: Grant/Research Support|Meissa Vaccines: Advisor/Consultant|Merck: Grant/Research Support|Moderna: Advisor/Consultant|Moderna: Grant/Research Support|Pfizer: Advisor/Consultant|Pfizer: Grant/Research Support|Sanofi Pasteur: Advisor/Consultant **Pedro A. Piedra, MD**, Ark Bioscience: Advisor/Consultant|Ark Bioscience: Grant/Research Support|GSK: Grant/Research Support|Icosavax: Advisor/Consultant|Icosavax: Grant/Research Support|Mapp Biologics: Grant/Research Support|Meissa Vaccines: Grant/Research Support|Moderna: Advisor/Consultant|Novavax: Advisor/Consultant|Novavax: Grant/Research Support|Sanofi-Pasteur: Grant/Research Support|Shionogi: Advisor/Consultant|Shionogi: Grant/Research Support|Takeda: Advisor/Consultant **Mary A. Staat, MD, MPH**, CDC: Grant/Research Support|Cepheid: Grant/Research Support|Merck: Grant/Research Support|NIH: Grant/Research Support|Pfizer: Grant/Research Support|Up-To-Date: Honoraria **John V. Williams, MD**, Merck: Grant/Research Support|Quidel: Board Member **Marian G. Michaels, MD, MPH**, Merck: Grant/Research Support|Viracor: Grant/Research Support

